# Retention vs. Stability: A Comparative Study of Thermoplastic Materials for Oral Appliance Therapy

**DOI:** 10.7759/cureus.110143

**Published:** 2026-06-02

**Authors:** Anna Shima, Hiroyuki Nakano, Naoki Tsukiyama, Ayano Nakada, Fukunori Toukai, Shintaro Kakuta, Yasuhisa Sawai, Takanobu Takata, Eiji Mitate, Satoshi Wada

**Affiliations:** 1 Department of Physical and Cognitive Rehabilitation Technology, Kanazawa Medical University Hospital, Kahoku, JPN; 2 Department of Oral and Maxillofacial Surgery, Kanazawa Medical University, Kahoku, JPN; 3 Department of Oral and Maxillofacial Surgery, Kanazawa Medical University, Ishikawa Pref, JPN; 4 Department of Pathology Ⅱ, Kanazawa Medical University, Kahoku, JPN

**Keywords:** obstructive sleep apnea (osa), oral appliances, retentive force, stability, thermoplastic materials

## Abstract

Background and aim

Retention and stability are important mechanical factors influencing the effectiveness of oral appliance therapy for obstructive sleep apnea (OSA). Retention refers to the force required to maintain the appliance in place, whereas stability reflects the ability to preserve retentive force during repeated use. Although thermoplastic materials are widely used for oral appliances, differences in their initial retention and long-term stability remain unclear. This study aimed to compare retentive force and stability over repeated removal cycles between two thermoplastic materials, Duran and New Duran, using standardized maxillary and mandibular models. We hypothesized that Duran would exhibit higher initial retentive force, whereas New Duran would demonstrate greater stability over repeated use.

Methods

Two types of thermoplastic materials were evaluated using standardized dental models. Retentive force was measured during 100 consecutive removal cycles. Each experiment was repeated three times. A linear mixed-effects model was used to analyze the effects of material type, removal cycle, and their interaction. Initial retentive force was compared using Welch’s t-test.

Results

In the maxillary model, Duran showed higher initial retentive force than New Duran, although the difference was not statistically significant (1.457 ± 0.117 N vs. 1.040 ± 0.251 N, p = 0.085). In contrast, in the mandibular model, Duran demonstrated significantly higher initial retention (1.683 ± 0.132 N vs. 0.857 ± 0.107 N, p = 0.0013). Over repeated removal cycles, Duran exhibited a gradual decrease in retention, whereas New Duran showed relatively stable retentive force. A significant interaction between material type and removal cycle was observed, indicating different degradation patterns between materials.

Conclusions

Duran provides a higher initial retentive force, particularly in the mandibular model, whereas New Duran demonstrates superior stability over repeated use. These findings suggest that material selection should be tailored according to anatomical conditions and clinical priorities.

## Introduction

Oral appliance (OA) therapy is widely used for the management of obstructive sleep apnea (OSA), particularly in patients with mild to moderate disease or those who are intolerant to continuous positive airway pressure (CPAP) therapy [1-6]. Previous studies and recent reviews have demonstrated that OA therapy can effectively reduce the apnea-hypopnea index and improve symptoms, thereby establishing it as an established alternative treatment modality [2-6]. Recent systematic and updated reviews have further supported the effectiveness of mandibular advancement devices and have suggested that appliance design may influence treatment outcomes [4,5].

The effectiveness of OA therapy depends not only on the degree of mandibular advancement but also on the ability of the appliance to maintain a stable mandibular position during sleep. Inadequate retention may result in appliance displacement, reduced mandibular advancement, and decreased therapeutic efficacy [7]. Therefore, both sufficient initial retention and long-term stability are important for successful OA therapy.

In clinical practice, thermoplastic materials are commonly used for OA fabrication. In this study, we evaluated the following two thermoplastic materials: Duran, a single-layer polyethylene terephthalate glycol-modified (PET-G) sheet, and New Duran, a dual-layer PET-G/thermoplastic polyurethane (TPU) sheet. The single-layer PET-G material is relatively rigid and may provide strong mechanical engagement, whereas the dual-layer PET-G/TPU material incorporates an elastic polyurethane layer and may offer improved resistance to deformation during repeated use [8,9].

In this study, “retention” refers to the magnitude of the retentive force required to dislodge the appliance, whereas “stability” refers to the ability to maintain retentive force over repeated removal cycles. Clarifying these two aspects is clinically relevant because a material with high initial retention may not necessarily maintain that retention during long-term use.

Furthermore, anatomical differences between the maxilla and mandible may influence retention mechanisms. The maxilla benefits from broader surface contact, whereas the mandible relies more heavily on mechanical engagement with the dentition [10]. These differences suggest that the performance of thermoplastic materials may vary depending on the jaw, yet this aspect has not been sufficiently investigated. In addition, the therapeutic effect of oral appliances may be influenced by the degree of mandibular protrusion, suggesting that stable retention is important for maintaining an appropriate mandibular position during sleep [11].

## Materials and methods

Materials and appliance fabrication

Two types of thermoplastic sheets were used for the fabrication of oral appliances. A single-layer polyethylene terephthalate glycol-modified (PET-G) thermoplastic sheet (Duran Plus, 1.5 × 125 mm; Chiyoda City, Japan: JM Ortho) and a dual-layer thermoplastic sheet composed of PET-G and thermoplastic polyurethane (TPU) (New Duran Soft, PD 1.8 × 125 mm; Chiyoda City, Japan: JM Ortho) were selected.

The direction of insertion and removal was determined using the working cast. Block-out and relief procedures were performed using a dental surveyor to standardize undercut conditions. Particular attention was given to the anterior teeth, especially the central and lateral incisors, which are single-rooted and more susceptible to excessive mechanical stress. To minimize the risk of overloading these teeth, relief was primarily applied to the anterior region, allowing occlusal forces to be redistributed to the premolar and molar regions. A duplicate cast was then fabricated using dental stone to serve as the final working model. To prevent damage to the cast during thermoforming, a thin separating foil (Isofolan foil, 0.1 × 125 mm) was adapted prior to sheet forming.

The trimming design of the appliances was standardized. In the maxillary model, the anterior region was trimmed to approximately half the crown height, whereas in the mandibular model, the trimming line followed the cervical margin. In both models, the posterior regions were trimmed at 2 mm below the cervical line. The border length was carefully adjusted to maintain uniformity among all specimens.

Measurement protocol

Retentive force was evaluated separately for the maxillary and mandibular models for each material. A custom attachment system was fabricated to ensure consistent force application during testing. A rigid polyvinyl chloride plate was first adapted to the dental arch, and a metal hook was attached at its center. The assembly was fixed using autopolymerizing resin, ensuring that the hook was aligned parallel to the base plane of the model.

**Figure 1:**
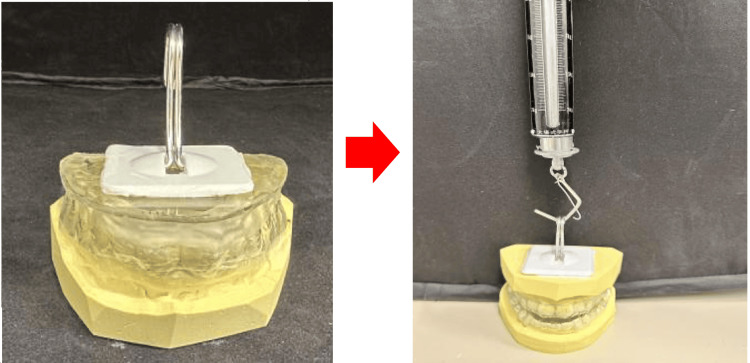
Measurement of retentive force using a custom attachment system. A rigid polyvinyl chloride plate was adapted to the dental arch, and a metal hook was attached to the center and fixed with auto-polymerizing resin. The hook was aligned parallel to the base plane of the dental cast to ensure consistent force application. Retentive force was measured using a tension gauge by applying a tensile load perpendicular to the base plane, simulating vertical dislodgement of the oral appliance from the maxillary or mandibular model.

After confirming proper alignment, the hook was securely attached to the appliance positioned on the dental cast. Retentive force was measured using a tension gauge (Tokyo, Japan: Ohba Keiki Co.). A tensile load was applied in a direction perpendicular to the base plane of the model to simulate vertical dislodgement (Figure 1).

The removal test was repeated for a total of 100 consecutive cycles. To minimize the influence of viscoelastic recovery, a 10 s interval was maintained between each cycle. Retentive force was recorded at every cycle, allowing for continuous evaluation of changes in retention during repeated insertion and removal.

This study was conducted as an in vitro experimental study and did not involve human participants or animals. Therefore, institutional review board approval was not required.

Statistical analysis

Continuous variables are presented as mean ± standard deviations. Initial retentive force was compared between PET-G and PET-G/TPU using Welch’s t-test separately for the maxillary and mandibular models, because equality of variance was not assumed. Changes in retentive force over repeated removal cycles were evaluated using linear mixed-effects models separately for the maxillary and mandibular models. In each model, material type, removal cycle, and the material × cycle interaction were included as fixed effects, and repeated measurements from each experimental series were treated as random effects. Exact p-values were reported, and p < 0.05 was considered statistically significant. All statistical analyses were performed using JMP Student Edition 18 (Cary, NC: SAS Institute Inc.).

## Results

Initial retentive force

The initial retentive force differed between materials and between jaw conditions. In the maxillary model, Duran showed higher initial retentive force than New Duran; however, the difference did not reach statistical significance (1.457 ± 0.117 N vs. 1.040 ± 0.251 N, p = 0.085).

Retentive force over repeated removal cycles

Maxillary Model

**Figure 2:**
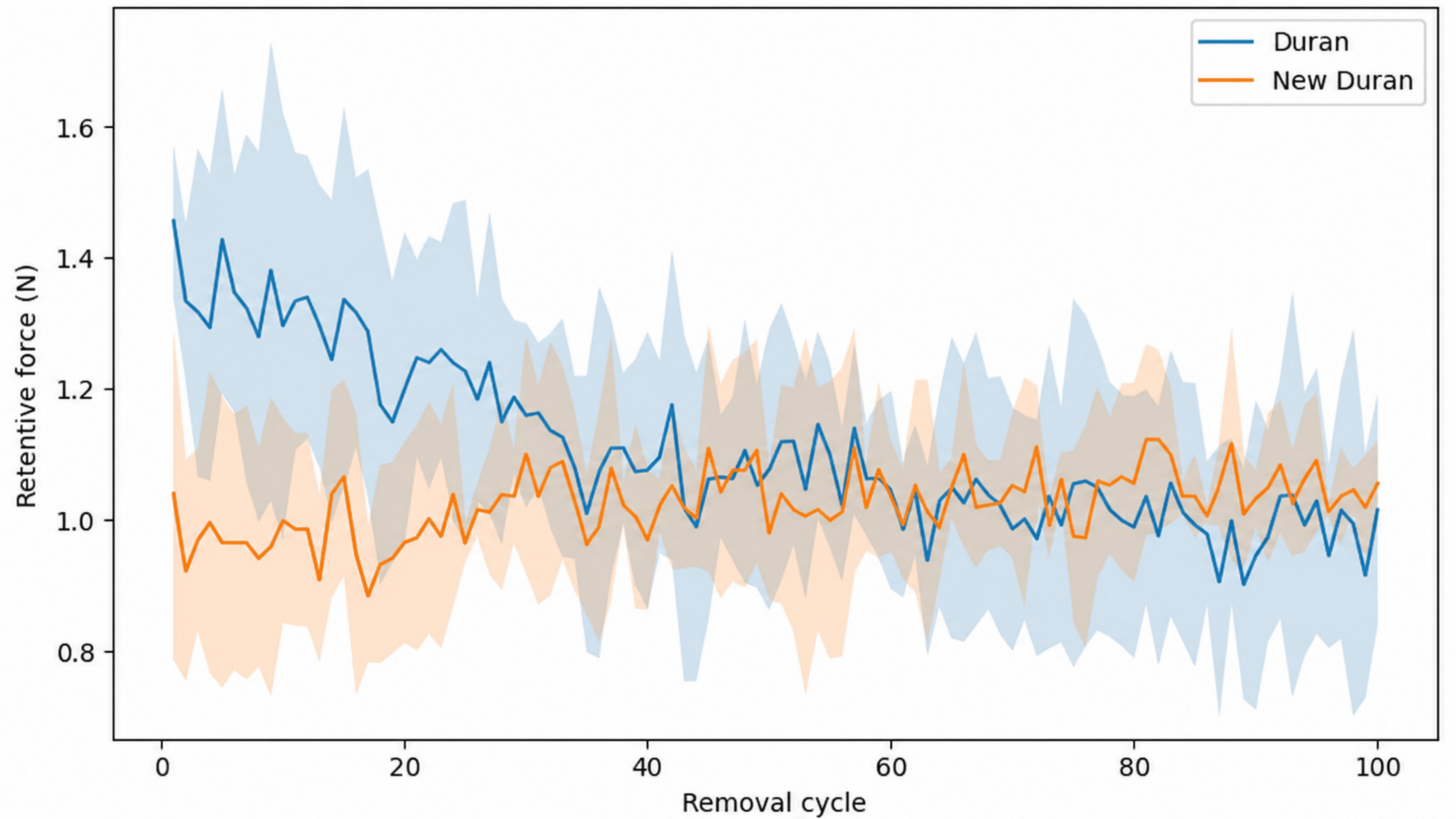
Retentive force over 100 removal cycles in the maxillary model. Duran showed higher initial retentive force, followed by a gradual decrease over repeated cycles. In contrast, New Duran maintained relatively stable retentive forces. Values are presented as mean ± standard deviation.

In the maxillary model, Duran exhibited a gradual decrease in retentive force with repeated removal cycles. Although the initial retentive force was higher in Duran, the difference between materials decreased over time. New Duran, on the other hand, showed relatively stable retentive force throughout the 100 removal cycles, with only minor fluctuations (Figure 2).

Mandibular Model

**Figure 3:**
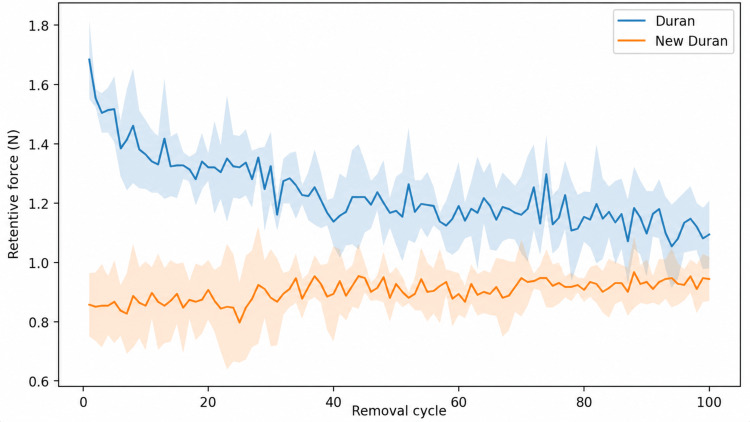
Retentive force over 100 removal cycles in the mandibular model. Duran demonstrated consistently higher retentive force than New Duran throughout the experiment. Although a gradual decrease was observed, Duran maintained superior retention after 100 cycles. Values are presented as mean ± standard deviation.

In the mandibular model, Duran consistently demonstrated higher retentive force than New Duran across all removal cycles. Although a gradual reduction in retentive force was observed, Duran maintained superior retention even after 100 cycles. New Duran showed lower retentive force overall but remained relatively stable across repeated cycles (Figure 3).

Comparison of retentive force between materials

The results of Welch’s t-test comparing the initial retentive force between PET-G and PET-G/TPU are summarized in Table 1. The difference between materials was not statistically significant in the maxillary model (p = 0.0846), whereas PET-G showed significantly higher initial retentive force than PET-G/TPU in the mandibular model (p = 0.0013).

**Table 1:** Welch’s t-test comparing initial retentive force. Values are presented as mean ± standard deviation. Retentive force is expressed in newtons (N). Welch’s t-test was used to compare the initial retentive force between PET-G and PET-G/TPU separately for the maxillary and mandibular models.

Model	PET-G (Duran), mean ± SD	PET-G/TPU (New Duran), mean ± SD	Test statistic	df	p-Value
Maxillary	1.46 ± 0.12 N	1.04 ± 0.25 N	t = 2.61	2.83	0.0846
Mandibular	1.68 ± 0.13 N	0.86 ± 0.11 N	t = 8.43	3.83	0.0013

Changes in retentive force over repeated removal cycles

The results of the linear mixed-effects model are summarized in Table 2. In both the maxillary and mandibular models, material type, removal cycle, and the material × cycle interaction were significantly associated with retentive force. In the maxillary model, the material × cycle interaction indicated that the difference between PET-G and PET-G/TPU changed over repeated removal cycles. In the mandibular model, PET-G maintained a higher retentive force than PET-G/TPU over the experimental period, although the pattern of change also differed between materials. These findings indicate that retentive force changed with repeated removal cycles and that the pattern of change differed between PET-G and PET-G/TPU.

**Table 2:** Linear mixed-effects model analysis of retentive force. Retentive force is expressed in newtons (N). A linear mixed-effects model was used to evaluate the effects of material type, removal cycle, and the material × cycle interaction on retentive force. Analyses were performed separately for the maxillary and mandibular models. Effect estimates are presented with standard errors, and exact p-values are shown.

Model	Fixed effect	Estimate, N	Standard error, N	Test statistic	df	p-value
Maxillary	Material type	-0.324	0.102	χ² = 10.12	1	0.0015
Maxillary	Removal cycle	-0.00386	0.00017	χ² = 495.30	1	<0.0001
Maxillary	Material × cycle interaction	0.00474	0.00025	χ² = 372.97	1	<0.0001
Mandibular	Material type	-0.541	0.057	χ² = 91.47	1	<0.0001
Mandibular	Removal cycle	-0.00333	0.00015	χ² = 485.77	1	<0.0001
Mandibular	Material × cycle interaction	0.00415	0.00021	χ² = 378.63	1	<0.0001

## Discussion

This study demonstrated that the retentive properties of thermoplastic materials used for oral appliance therapy differ not only in magnitude but also in temporal stability and jaw-dependent performance. Specifically, Duran exhibited higher initial retentive force, whereas New Duran showed greater stability over repeated removal cycles. Notably, the pattern of retention change differed between the maxillary and mandibular models, highlighting the importance of considering anatomical conditions when selecting materials for oral appliances.

The progressive reduction in retentive force observed with repeated removal cycles suggests that greater rigidity may predispose the material to deformation or loss of adaptation under cyclic loading. Similar reductions in retention associated with repeated use have been reported in previous studies of oral appliances and thermoplastic materials [7-9].

Retention is a critical determinant of the therapeutic efficacy of oral appliances in patients with obstructive sleep apnea, as insufficient retention may lead to appliance displacement and reduced mandibular advancement during sleep. Previous studies have emphasized that mandibular repositioning appliances are effective only when an appropriate mandibular position is maintained, highlighting the importance of both mechanical engagement and long-term stability for treatment effectiveness [3,11].

Previous studies have emphasized that both mechanical engagement and long-term stability are essential for maintaining treatment effectiveness [3]. In addition, the therapeutic effect of oral appliances may be influenced by the degree of mandibular protrusion, suggesting that stable retention is important for maintaining an appropriate mandibular position during sleep [11]. However, the progressive reduction in retentive force observed with repeated removal cycles suggests that such rigidity may also predispose the material to deformation or loss of adaptation under cyclic loading. Similar reductions in retention associated with repeated use have been reported in previous studies of oral appliances and thermoplastic materials [7-9].

In contrast, New Duran, composed of a dual-layer structure incorporating thermoplastic polyurethane (TPU), demonstrated lower initial retention but greater stability over time. The elastic properties of TPU likely contribute to improved recovery following deformation, thereby maintaining retentive force during repeated cycles. This viscoelastic behavior has been recognized as a key factor influencing the long-term performance of polymer-based dental materials [8,9].

A particularly important finding of this study is the difference in material performance between the maxilla and mandible. In the maxillary model, the difference in retentive force between materials decreased over time, suggesting that stability may be more relevant than initial retention in this region. This may be explained by the presence of additional retention mechanisms in the maxilla, such as suction and broader surface contact [7]. In contrast, the mandibular model showed a persistent advantage of Duran, indicating that mechanical retention plays a more dominant role in this region.

In addition to these functional differences, anatomical variations in tooth morphology between the maxilla and mandible may further contribute to the observed differences in retentive force. The maxillary dentition generally presents with broader crown morphology and more favorable undercut distribution, which enhances adaptation and provides auxiliary retention through surface contact and suction effects. Conversely, mandibular teeth tend to exhibit narrower crown forms and reduced undercut engagement, leading to a greater dependence on direct mechanical retention. Therefore, even when the same thermoplastic material is used, differences in tooth morphology and undercut characteristics may result in significant variations in retentive force between the maxillary and mandibular models [7,10]. These findings emphasize that material performance should be interpreted in the context of anatomical conditions rather than in isolation.

Long-term oral appliance therapy may also be associated with dental and occlusal changes, including changes in overjet, overbite, and tooth position. Therefore, material selection should be considered not only from the perspective of initial retentive force but also in relation to long-term mechanical stability and potential dental side effects [12-15].

From a clinical perspective, these results suggest that material selection for oral appliance therapy should be individualized. Duran may be advantageous in cases requiring strong initial retention, particularly in mandibular appliances where mechanical engagement is critical. Conversely, New Duran may be preferable in situations where long-term stability and resistance to material fatigue are prioritized, such as in patients who frequently remove and insert the appliance. This concept aligns with recent trends toward personalized treatment strategies in sleep medicine and dental device therapy [2,3,6].

However, the clinical application of jaw-specific material selection should be interpreted with caution. In clinical practice, oral appliances for obstructive sleep apnea are commonly fabricated as monoblock or two-block devices, and the use of different thermoplastic materials for opposing jaws within a single appliance may not always be technically feasible or routinely available in dental laboratories. This is particularly relevant when comparing materials with different structures, such as single-layer PET-G and dual-layer PET-G/TPU sheets. Therefore, the present findings should not be interpreted as a direct recommendation to combine different materials for the maxillary and mandibular components in all cases. Rather, they provide material-specific mechanical information that may help clinicians and technicians select materials according to appliance design, jaw-dependent retention requirements, and clinical priorities.

This study has several limitations. First, the in vitro design does not fully replicate intraoral conditions, including the effects of saliva, temperature changes, and biological adaptation. Second, the number of experimental repetitions was limited, which may have affected statistical power, particularly in the maxillary analysis. Third, only vertical dislodgement forces were evaluated, whereas clinical conditions involve more complex multidirectional forces.

Future research should include clinical studies to validate these findings in vivo. In addition, further investigation into the mechanical behavior of thermoplastic materials under cyclic loading conditions, including creep and fatigue properties, would provide valuable insights. Advanced analytical techniques, such as finite element analysis, may also help clarify the biomechanical mechanisms underlying retention and material degradation [13,14].

## Conclusions

Duran provided higher initial retentive force, particularly in the mandibular model, whereas New Duran demonstrated superior stability over repeated use. These findings suggest that thermoplastic materials have different mechanical characteristics depending on the jaw model and repeated removal conditions. Although practical constraints in appliance fabrication should be considered, these results may provide useful information for material selection according to appliance design, anatomical conditions, and clinical priorities.
